# Prohibiting alcohol sales during the coronavirus disease 2019 pandemic has positive effects on health services in South Africa

**DOI:** 10.4102/phcfm.v12i1.2528

**Published:** 2020-07-15

**Authors:** Hermann Reuter, Louis S. Jenkins, Marischka De Jong, Steve Reid, Michael Vonk

**Affiliations:** 1 Primary Health Care Directorate, Faculty of Health Sciences, University of Cape Town, Cape Town, South Africa; 2 Department of Family and Emergency Medicine, Division of Family Medicine and Primary Care, Faculty of Medicine and Health Sciences, Stellenbosch University, Cape Town, South Africa; 3 George Hospital, Garden Route District, Western Cape Department of Health, George, South Africa; 4 Department of Family and Emergency Medicine, George Hospital, Garden Route District, Western Cape Department of Health, George, South Africa

**Keywords:** COVID-19, alcohol, restrictions, trauma, emergency centres, reductions

## Abstract

As the coronavirus disease 2019 (Covid-19) pandemic evolves globally, we are realising its impact on communities from the disease itself and the measures being taken to limit infection spread. In South Africa (SA), 62 300 adults die annually from alcohol-attributable causes. Alcohol-related harm can be reduced by interventions, such as taxation, government monopolising retail sales, outlet density restriction, hours of sales and an advertising ban. To mitigate the impact of the Covid-19 pandemic, SA instituted a lockdown that also prohibited alcohol sales. This led to a sharp reduction in unnatural deaths in the country from 800–1000/week to around 400/week during the lockdown. We reviewed three 2-week periods at a large rural regional hospital: Before Covid-19 (February), during social distancing (March) and during lockdown with alcohol ban (April). A dramatic drop in patient numbers from 145 to 64 (55.8%) because of assault, from 207 to 83 (59.9%) because of accidents, from 463 to 188 (59.4%) because of other injuries and from 12 to 1 (91.6%) because of sexual assaults was observed during the first 2 weeks of lockdown. As healthcare professionals, we need to advocate for the ban to remain until the crisis is over to ensure that health services can concentrate on Covid-19 and other patients. We encourage other African states to follow suit and implement alcohol restrictions as a mechanism to free up health services. We see this as an encouragement to lobby for a new normal around alcohol sales after the pandemic. The restrictions should focus on all evidence-based modalities.

## Background

As the coronavirus disease 2019 (Covid-19) pandemic evolves globally, we are realising its impact on communities because of the disease itself and the measures being taken to limit infection spread. In South Africa (SA), 62 300 adults die annually from alcohol-attributable causes.^1^ Strong evidence exists that alcohol policy has a major impact on controlling alcohol consumption. The World Health Organization (WHO) lists the five most effective policy interventions: alcohol taxes, government monopolies for retail sale, restrictions on outlet density, restrictions on days and hours of sale and complete ban on advertising.^2^

Many African countries have various levels of restriction of alcohol consumption through policies. A study comparing four regulatory categories (price, availability, marketing and drink-driving) demonstrated a significant correlation between increased restrictiveness amounting to reduced alcohol per capita consumption.^3^ In this study of 46 African countries, South Africa ranked 14th lowest in terms of restrictions on alcohol, whilst Algeria ranked the highest, with a consequently very low alcohol consumption.^3^ In SA, we have seen the density of drinking establishments increasing and times of sales relaxing leading to increased alcohol consumption. The Liquor Amendment Bill of 2017, meant to address this deficiency, is stalled in parliament.^4^

## Coronavirus disease 2019, lockdown and alcohol restrictions

On 05 March 2020, the first case of Covid-19 was confirmed in SA. President Ramaphosa declared a state of disaster on 15 March, enforcing a travel ban, social distancing and closure of schools and universities. A national lockdown came into effect on 26 March that restricted all but essential services staff to stay at home and only leave home to access health care or buy essential items. Alcohol was classified as non-essential, effecting a total ban on legal sales of alcohol. All this led to no social gatherings, empty streets, no public alcohol drinking and emptier emergency centres (ECs) and morgues. The Southern African Alcohol Policy Alliance (SAAPA), with affiliates in Malawi, Zambia, Madagascar, Lesotho, Botswana, Namibia, Zimbabwe and SA, supports the increased restriction of alcohol availability, in line with the WHO Global Strategy to Reduce Alcohol Harm.^5^ Prior to Covid-19, as much as 30% of hospital admissions were alcohol-related.^5^

## Health benefits

**FIGURE 1:**
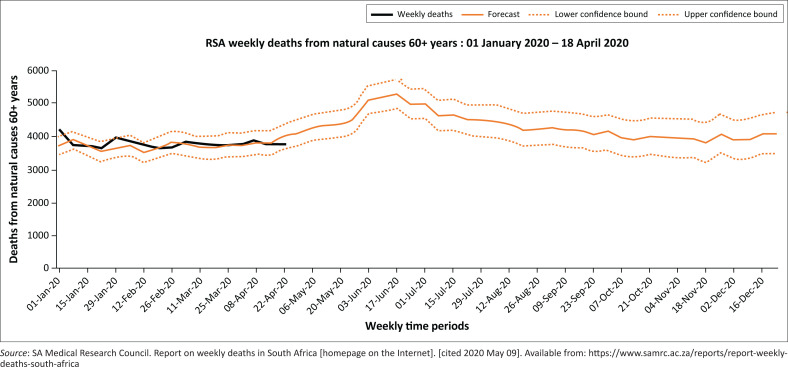
Weekly deaths from natural causes in South Africa (01 January–28 April 2020).

**FIGURE 2:**
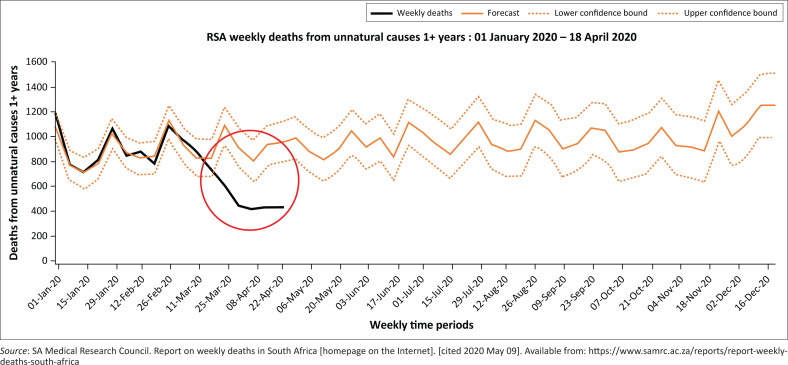
Weekly deaths from unnatural causes in South Africa (01 January–28 April 2020).

The ban on alcohol sales has shown striking health benefits and valuable lessons for regulation. There has been fewer alcohol-attributable hospital admissions. For example, Groote Schuur Hospital in Cape Town reported 66% fewer trauma admissions. Whist other factors, such as the restrictions on travel, less traffic and lockdown probably contributed, the limitations placed on the binge drinking culture in SA, often in drinking outlets close to people’s homes, with the associated violent engagements that often accompany it, most likely also played a significant role.^5^ There has also been a reduction in contact crime, for example, attempted murder cases from 1300 to 443, rape cases from 2908 to 371, assault cases from 11 876 to 1758 and domestic violence cases decreased by 69.4%.^5^ Whilst these outcomes cannot be attributed to the ban on alcohol sales only, the impact at least suggests some association.^5^ Domestic violence is difficult to measure, as it depends on reporting. It seems that initially, with less alcohol available, a reduction is noted, whilst it remains to be seen what the effects of lockdown will be. The number of unnatural deaths as recorded in the national register has shown a dramatic reduction, whilst deaths from natural causes have remained constant^6^ (see Figures 1 and 2).

## Impact on the George Hospital Emergency Centre

George Regional Hospital is a 272-bed rural public hospital in the Garden Route district of the Western Cape Province in SA. It is situated on the coastline 440 km east of Cape Town and manages 4000 emergency patients monthly and with the clinics care for 84% of the local community of 210 000 people. Three separate 2-week periods were reviewed from data captured in the electronic patient registration system: Before Covid-19 (February), after the national Disaster Declaration (March) and after lockdown with alcohol ban (April). A dramatic drop in patient numbers from 145 to 64 (55.8%) because of assault, from 207 to 83 (59.9%) because of accidents, from 463 to 188 (59.4%) because of other injuries and from 12 to 1 (91.6%) sexual assaults was observed during the first 2 weeks of lockdown. This was in line with national figures. A specific look at road-related incidents to see if the decrease was because of less cars being on the road revealed that they only made up a minor fraction of injuries: Of the 207 patients recorded as road traffic injuries in 2 weeks in February, the subset caused by road traffic incidents was 34 (16 pedestrians, 12 car occupants, four motorcyclists and two cyclists). In 2 weeks in April during the lockdown, of the 83 recorded incidents, six happened on the road (five car occupants and one pedestrian) (see Table 1).

**TABLE 1:** Impact of lockdown and alcohol ban on the George Hospital Emergency Centre.

Category	February	March	April
Date	02-02-2020–16-02-2020	08-03-2020–22-03-2020	29-03-2020–12-04-2020
Assaults	142	154	64
Road traffic incidents	207	207	83
Injuries	463	484	188
Sexual assaults	12	6	1

**FIGURE 3:**
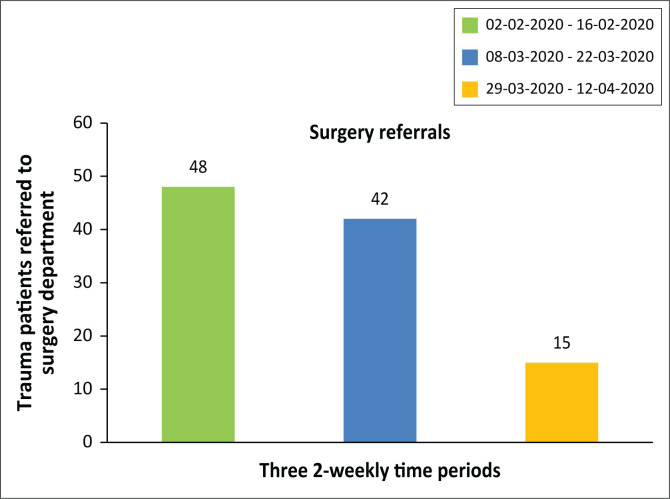
Referrals of trauma patients to the Surgery Department at George Hospital.

**FIGURE 4:**
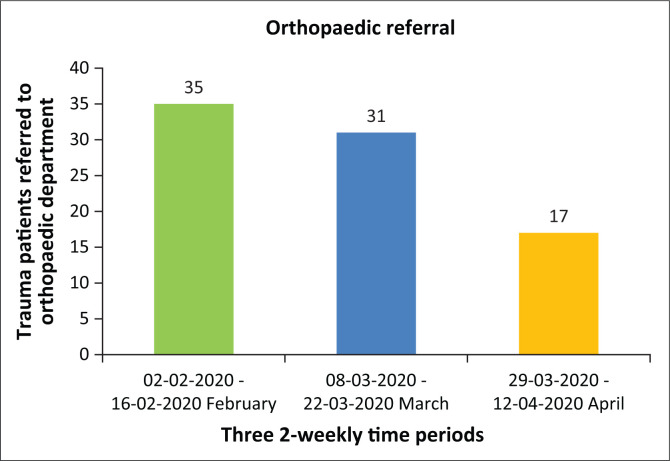
Referrals of trauma patients to the Orthopaedic Department at George Hospital.

A review of trauma referrals to the surgery and Orthopaedic departments during these three periods showed a similar sharp reduction (see Figures 3 and 4).

### Ethical consideration

This article followed all ethical standards for a research without direct contact with human or animal subjects.

## Discussion

This cross-sectional review of data from an EC in a large public hospital in SA has shown that the Covid-19 lockdown and alcohol ban have had a significant impact on trauma-related numbers. Whilst this was not a formal study, recognising the possibility of confounders, the association between alcohol and travel ban and the reduction of EC trauma-related attendees cannot be ignored. A 2017 Cape Town study found 1045 outlets, with 11% trading legally and people living within 3–5 min walking distance from their nearest alcohol outlet.^7^ Research shows that easy access to alcohol encourages consumption.^5^ If alcohol remained banned but the restriction on social gatherings was lifted, we suspect that the incidence of trauma, including road traffic accidents, would remain low. If alcohol was made available again, but people continued to be restricted in terms of social gathering, we suspect that the incidence would rise again. Of course, historically, the counter argument has been that with prohibition the so-called black market in alcohol sales flourishes. This complex issue needs addressing on a global scale.^8^ The government has demonstrated that it can act in the interest of public health. Recognising that alcohol restrictions have many economic implications, the regulation of alcohol requires a reactivation of the Inter-Ministerial Committee on Substance Abuse, the passing of the 2017 Alcohol Bill^4^ and a whole of society approach to improve the healthcare of the people of SA.^9^

## Conclusion

With Covid-19 causing upheaval internationally, healthcare workers face many uncertainties. One certainty is that a prohibition of alcohol sales in South Africa has reduced pressure on emergency care units and lowered mortality. Healthcare professionals need to lobby for restrictions to remain until the crisis is over to ensure that health services can concentrate on dealing with Covid-19 and other patients. We encourage other African states to follow suit and implement alcohol restrictions as a mechanism to free up health services. Additionally, this is an encouragement to lobby for a new normal around alcohol sales post-pandemic. The restrictions should focus on all evidence-based modalities, including increasing alcohol taxes, restricting outlets density, reducing opening times, banning advertising and increasing legal drinking age to 21 years.^10^
